# Racial inequities and rare *CFTR* variants: Impact on cystic fibrosis diagnosis and treatment

**DOI:** 10.1016/j.jcte.2024.100344

**Published:** 2024-04-20

**Authors:** Malinda Wu, Jacob D. Davis, Conan Zhao, Tanicia Daley, Kathryn E. Oliver

**Affiliations:** a Department of Pediatrics, Emory University School of Medicine, Atlanta, GA, USA; b Pediatric Institute, Children’s Healthcare of Atlanta, Atlanta, GA, USA; c Department of Biomedical Engineering, Georgia Institute of Technology and Emory University, Atlanta, GA, USA; d Interdisciplinary Graduate Program in Quantitative Biosciences, Georgia Institute of Technology, Atlanta, GA, USA

**Keywords:** Cystic fibrosis, African, Cystic fibrosis-related diabetes, Diagnosis, Newborn screening, Genetics

## Abstract

Cystic fibrosis (CF) has been traditionally viewed as a disease that affects White individuals. However, CF occurs among all races, ethnicities, and geographic ancestries. The disorder results from mutations in the *CF transmembrane conductance regulator* (*CFTR*). Varying incidence of CF is reported among Black, Indigenous, and People of Color (BIPOC), who typically exhibit worse clinical outcomes. These populations are more likely to carry rare *CFTR* variants omitted from newborn screening panels, leading to disparities in care such as delayed diagnosis and treatment. In this study, we present a case-in-point describing an individual of Gambian descent identified with CF. Patient genotype includes a premature termination codon (PTC) (c.2353C>T) and previously undescribed single nucleotide deletion (c.1970delG), arguing against effectiveness of currently available CFTR modulator-based interventions. Strategies for overcoming these two variants will likely include combinations of PTC suppressors, nonsense mediated decay inhibitors, and/or alternative approaches (e.g. gene therapy). Investigations such as the present study establish a foundation from which therapeutic treatments may be developed. Importantly, c.2353C>T and c.1970delG were not detected in the patient by traditional *CFTR* screening panels, which include an implicit racial and ethnic diagnostic bias as these tests are comprised of mutations largely observed in people of European ancestry. We suggest that next-generation sequencing of *CFTR* should be utilized to confirm or exclude a CF diagnosis, in order to equitably serve BIPOC individuals. Additional epidemiologic data, basic science investigations, and translational work are imperative for improving understanding of disease prevalence and progression, *CFTR* variant frequency, genotype-phenotype correlation, pharmacologic responsiveness, and personalized medicine approaches for patients with African ancestry and other historically understudied geographic lineages.

## Introduction

Cystic fibrosis (CF) is a lethal, autosomal recessive disorder that arises from loss-of-function variants in the *CF transmembrane conductance regulator* (*CFTR*). Respiratory failure remains the most prevalent cause of mortality, although people with CF also experience osteopenia [1], intestinal malabsorption [2], exocrine pancreatic insufficiency [3], and cystic fibrosis-related diabetes (CFRD) [4], among other comorbidities. The disease has been historically understudied in individuals with ancestral origins linked to non-European countries [5]. During the first nine years (2010–2018) of CF newborn screening (NBS) in the U.S., infants diagnosed with CF were 6–7% African American/Black, 13% Hispanic/Latin ethnicity (any race), and 4–5% some other race [6]. Prevalence of CF among non-White demographics has remained constant, with more recent data showing the U.S. CF population is approximately 5% African American/Black, 9.1% Hispanic/Latin (any race), and 4.2% other lineages [7]. However, these groups are frequently unrepresented in clinical trials [8], and suffer worse overall outcomes than White counterparts [9], [10], [11], [12], [13].

Furthermore, people with minoritized geographic ancestries often carry *CFTR* variants excluded from mutation panel(s) employed by CF NBS programs [14], [15], [16]. Examples include “nonsense” variants (categorized as CFTR “class I” defects), which prevent synthesis of full-length CFTR protein through introduction of a premature termination codon (PTC). These mutations are generally insensitive to correction by FDA-approved small molecules designed to restore CFTR function [17], [18], [19], [20], leaving many Black, Indigenous, or People of Color (BIPOC) without effective therapeutic options to address their underlying genetic defects [21]. In the present study, we discuss a case-in-point illustrated by a Gambian individual with CF, who harbors two class I *CFTR* variants: c.2353C>T and c.1970delG – the latter of which is a novel mutation reported here for the first time. These two variants were not detected by traditional *CFTR* screening panels, but instead through *CFTR* next-generation sequencing (NGS). Importantly, both c.2353C>T and c.1970delG are not on-label for currently available CFTR modulators. What follows is a detailed report of the patient’s first 12 years of life, together with a recent health status update at age 15 years.

### Patient presentation

#### Initial screening

In Washington state, an African American/Black female was born via an uncomplicated vaginal delivery at 36 weeks gestation. Meconium passed on post-natal day zero. The patient presented with testing concerning for CF as evidenced by a positive NBS (immunoreactive trypsinogen 180.7 ng/mL). Sweat chloride evaluation occurred at six weeks of age (97 mmol/L). Family history did not include any known relatives with CF. A few months prior to the patient’s birth, the family emigrated from Gambia to Washington state. Both parents reported that all known family lineages trace back to Gambia. One exception was a single set of maternal great-grandparents, who were from Mali.

#### Diagnosis and genotyping

**Fig. 1:**
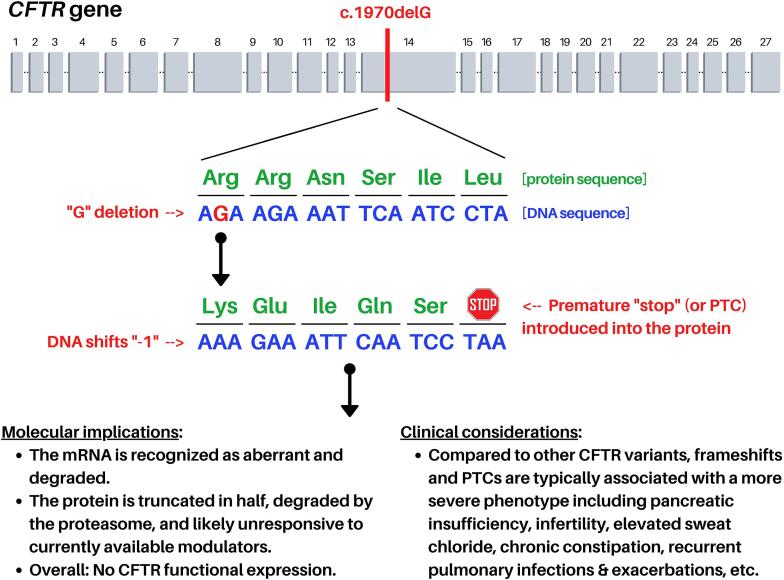
**Consequences of c.1970delG in *CFTR***. At *CFTR* chromosomal position 1970 (c.1970), deletion of the G nucleotide (delG) confers a DNA frameshift together with premature termination of the protein. Regions of *CFTR* DNA that encode protein (i.e. exons) are annotated as grey boxes and numbered. For interpretation of the references to color in this figure legend, the reader is referred to the web version of this article.

A CF diagnosis was not conferred until age 10 months (m) following multiple tiers of *CFTR* DNA analysis. Because the Washington NBS program did not incorporate *CFTR* variant evaluation until 2019, DNA testing was outsourced to Eurofins (Tucker, Georgia, USA). An initial panel screen for 23 “common” *CFTR* variants recommended by the American College of Medical Genetics and Genomics (ACMG) [22] was conducted and failed to identify any mutations. Subsequent *CFTR* exome sequencing of all coding exons and immediate flanking regions was performed by Eurofins, which detected single copies of two protein-coding defects: c.2353C>T and c.1970delG. According to the genetic report, these variants are likely severe/pathogenic as c.2353C>T results in a PTC (p.Arg785Ter or R785X), and c.1970delG elicits a frameshift with downstream PTC (p.Arg657LysfsX5) (Fig. 1). Moreover, both mutations are not included among the 25 *CFTR* variants classified as non-disease causing [23], [24]. The patient was also identified as heterozygous for a synonymous single nucleotide polymorphism, c.2562T>G (p.Thr854Thr or T854T), and homozygous for two intronic repeat alleles, c.744-33GATT and c.1210–12T. Nucleotide numbering is based on GenBank accession number NM_000492. See Table 1 for a glossary of genetic terms utilized in this report.

**Table 1:** Genetic terms utilized in the present study.

**Term**	**Definition**	**References**
**Genotype**	An individual's collection of genes. The term can also refer to the two alleles inherited for a particular gene.	[100]
**Phenotype**	An individual's observable traits, which may be determined by environmental factors and/or genetic contributions (i.e. genotype).	[100]
**Chromosome**	An organized package of DNA found in the nucleus of the cell. Humans possess 23 pairs of chromosomes: 22 pairs of numbered chromosomes (autosomes) and one pair of sex chromosomes (X and Y).	[100]
**Autosomal recessive**	Pattern of inheritance characteristic of some genetic diseases. “Autosomal” refers to the gene of interest being located on one of the numbered (non-sex) chromosomes. “Recessive” indicates that two copies of a variant are necessary to cause disease.	[100]
**Allele**	One of two or more versions of a gene. An individual inherits two alleles for each gene, one from each parent. If the two alleles are the same, the individual is homozygous for that gene. If the alleles are different, the individual is heterozygous.	[100]
**Nucleotide**	Basic building block of nucleic acids. A nucleotide consists of a sugar molecule (deoxyribose in DNA or ribose in RNA) attached to a phosphate group and a nitrogen-containing base. The bases present in DNA are adenine (A), cytosine (C), guanine (G), and thymine (T). In RNA, uracil (U) replaces thymine.	[100]
**Base Pair**	Two bases bonded to one another in a DNA helix. DNA consists of two strands twisted around each other. The two strands are held together by hydrogen bonds between the bases, with adenine pairing to thymine and cytosine pairing to guanine.	[100]
**Variant**	A permanent change in a gene’s DNA sequence that is different from what is considered the normal or “wild-type” sequence. Variants range in size from a single nucleotide to a large segment of chromosome. Variants can occur in three ways: (1) inheritance from a parent; (2) random emergence during egg or sperm formation; or (3) acquired during a person's lifetime. If a specific non-deleterious DNA change is present in at least 1% of the population, it is called a polymorphism. If the DNA change causes an abnormal protein, then it is referred to as a variant.	[24]
**CFTR class I mutation**	A category of CFTR variants that exhibit defect(s) in production of RNA and/or full-length protein. These may include premature termination codons (see definition below), insertions/deletions (extra DNA bases are either added or missing, respectively), or splice variants (different portions of the gene are put together incorrectly), among others.	[24], [101], [102]
**Premature termination codon (PTC; or “nonsense” mutation)**	Substitution of a single base pair that leads to appearance of a stop codon where there was a codon previously specifying an amino acid. Presence of this premature termination codon results in the production of a shortened, and likely nonfunctional protein.	[100]
**Frameshift**	A type of mutation involving the insertion or deletion of a nucleotide, whereby the resulting inserted or deleted base pairs are not divisible by three. Genes are decoded in groups of three bases. Each group of three bases corresponds to one of 20 different amino acids used to build a protein. If a mutation disrupts this reading frame, then the entire DNA sequence downstream the mutation will be read incorrectly.	[100]
**Synonymous (or “silent”) single nucleotide polymorphism**	A type of polymorphism in which a single base pair is mutated, but the altered codon corresponds to the same amino acid in the normal or “wild-type” sequence.	[100]
**Exon**	The portion of a gene that encodes amino acids and is therefore expressed in the protein product. In higher eukaryotes, most exons are separated by one or more non-coding DNA sequences termed introns. These segments are not expressed in the protein as they are excised out.	[100]
**Intron**	The portion of a gene that does not encode amino acids.	[100]
**Targeted (or “traditional”) CFTR screening**	Screening performed using a panel of 23 CFTR variants recommended by the American College of Medical Genetics. These variants have been shown to cause CF, both through clinical and laboratory evaluation. The list represents many of the most frequently observed CFTR variants in U.S. populations of European descent.	[22], [24], [78]
**Next-generation sequencing**	A high-throughput method used to determine the exact sequence of bases (A, C, G, T) in a DNA molecule. The technique is capable of processing multiple DNA strands in parallel to sequence an individual’s genome, for example. The two most common approaches include: whole exome sequencing, which only covers protein-coding regions (i.e. exons); and (2) whole genome sequencing, which covers both exons and non-protein coding regions (i.e. introns).	[100]
**CFTR modulators**	Small molecules that correct the underlying genetic defect(s) caused by CFTR variants. These drugs are clinically approved in several countries for specific variants, or groups of variants, that introduce abnormalities in CFTR functional expression.	[18], [24], [101], [103]
**c.2562T>G (p.Thr854Thror T854T)**	A CFTR variant at position 2562 in the gene’s DNA, in which thymine is mutated to guanine. In the corresponding protein sequence at position 854, the encoded “wild-type” amino acid, threonine (Thr) remains unaltered. Therefore, this variant is classified as a synonymous single nucleotide polymorphism.	[24], [104], [105]
**c.2353C>T (p.Arg785Ter or R785X)**	A CFTR variant at position 2353 in the gene’s DNA, in which cytosine is mutated to thymine. In the corresponding protein sequence at position 785, the encoded “wild-type” amino acid, arginine (Arg), is replaced by a premature termination codon (X). Therefore, this variant is classified as a nonsense mutation.	[24], [106]
**c.1970delG (p.Arg657LysfsX5)**	A CFTR variant at position 1970 in the gene’s DNA, in which a single guanine nucleotide is deleted and therefore causes a frameshift (fs). In the corresponding protein sequence at position 657, the encoded “wild-type” amino acid, arginine (Arg), is changed to lysine (Lys). In addition, a premature termination codon is introduced five amino acids downstream (X5). Therefore, this variant is classified as a frameshift mutation.	This study

#### Pulmonary phenotype

**Fig. 2:**
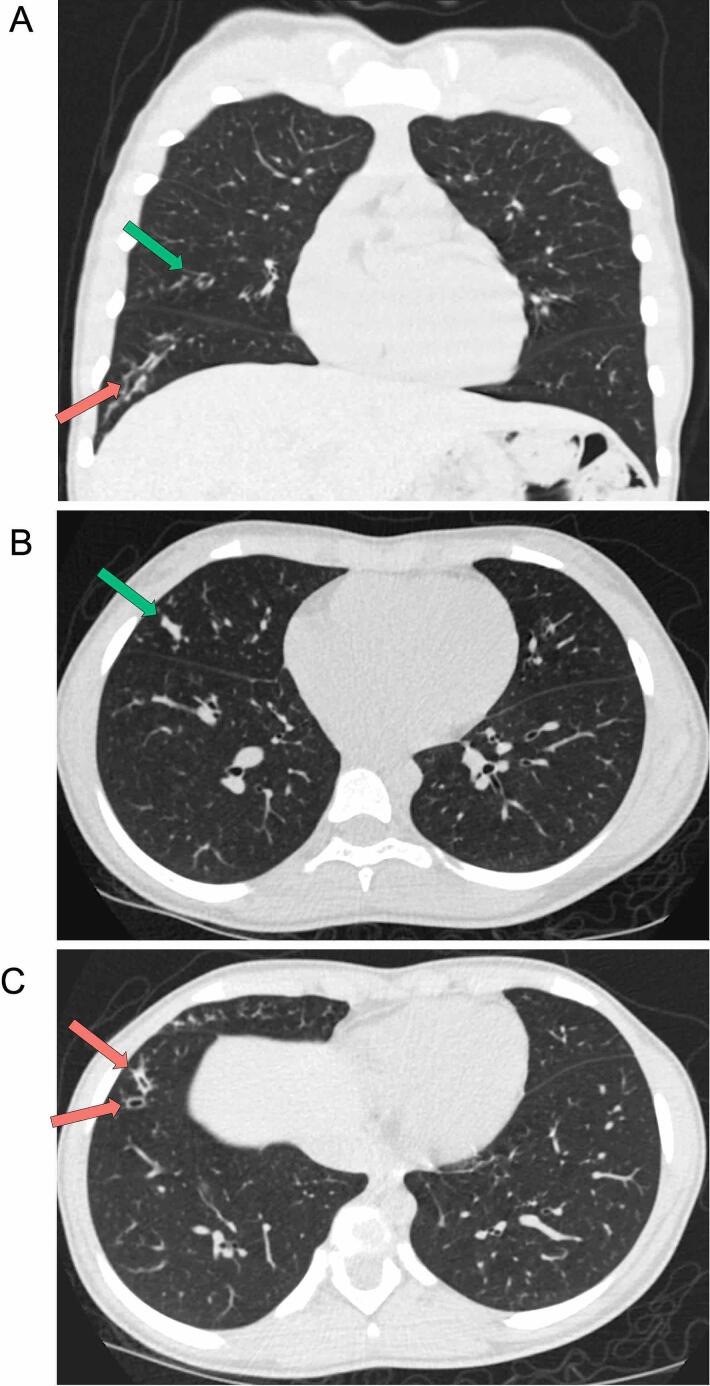
**Chest CT at 11.8y identifies pathological respiratory features.** Mild bronchiectasis (right lower lobe, red arrows) and mucus plugging (right middle lobe, green arrows) are demonstrated on coronal (A) and axial (B-C) views. For interpretation of the references to color in this figure legend, the reader is referred to the web version of this article.

The patient was first hospitalized at age 4m with bronchiolitis and an acute pulmonary exacerbation (APE) compounded by reactive airway disease. This event triggered initiation of pulmonary treatments described in Table 2. Five subsequent APEs requiring hospitalization occurred at ages 3.2 years (y), 5.3y, 9.2y, 9.8y, and 10.7y. During each APE, treatment included antibiotics, chest physiotherapy, and/or oral steroids. A routine chest CT at age 11.8y identified mild cylindrical bronchiectasis and mucus plugging (Fig. 2). Recent measurements of percent predicted forced expiratory volume in one second (FEV1) exhibited the following decline: 95% (10y), 91% (11y), 92.75% (12y), 86.75% (13y), 83% (14y), 72% (15y).

**Table 2:** **Summary of clinical findings and interventions.** Occurrence of an individual treatment is annotated as acute (a), daily (d), or weekly (w). Administration frequency is indicated as (x).

**Organ system**	**Manifestation**	**Age(s) of occurrence**	**Treatment**
**Pulmonary**	Bronchiolitis	4m	− Albuterol (d; 2x) − 7% hypertonic saline (d; 2x) − Dornase alfa (d; 1x) − Chest physiotherapy (d; 2x)
	APE	4m, 3.2y, 5.3y, 9.2y, 9.8y, 10.7y	− Antibiotics (a) − Oral steroids (a) − All interventions listed above for Bronchiolitis
	Cylindrical bronchiectasis	11y	− Azithromycin (w; 3x)
**Endocrine**	Abnormal HbA1C	4y	− None
	CFRD	6y	− Insulin (d; 4x or more)
	Vitamin D deficiency	7y	− Vitamin D3 (w; 1x)
**Gastrointestinal**	Pancreatic insufficiency	2m	− Enzyme replacement (d; with food) − Multivitamins (d)
	Chronic constipation	2m	− Osmotic laxatives (d; 1x)
	Transaminitis	3y	− Resolved without intervention
	Appendicitis	6y	− Appendectomy (a)
**Musculoskeletal**	Scoliosis	9y	− Monitored without intervention
**Cardiovascular**	Deep vein thrombosis	9.8y	− Subcutaneous enoxaparin (a)
**Sinus**	Chronic sinusitis with polyps	11y	− Polypectomy (a) − Intranasal steroid (d; 1x) − Sinus rinse (d; 1x) − Oral antihistamine (d; 1x)
**Neurological**	Restless leg syndrome	12y	− Iron (d; 1x)

#### Microbiology

**Fig. 3:**
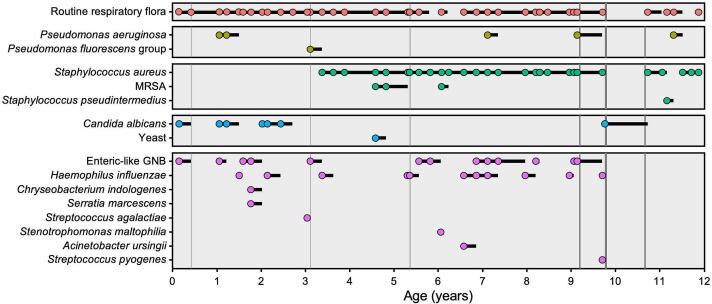
**Microbial and related pulmonary manifestations over time**. Pathogenic cultures show varying microbial taxa since birth. Solid black horizontal lines indicate inferred microbial presence. Vertical grey bars denote APEs (six in total), with line thickness representing length of hospitalization. MRSA, methicillin-resistant *Staphylococcus aureus*; GNB, Gram-negative bacillus. For interpretation of the references to color in this figure legend, the reader is referred to the web version of this article.

Repetitive *Pseudomonas aeruginosa*, methicillin-resistant *Staphylococcus aureus* (MRSA), and *Candida albicans* infections have been noted (Fig. 3). Infections of *P. aeruginosa* initiated at age 12m and recurred at ages 7.1y, 9.2y, and 11.3y – all of which were cleared with nebulized tobramycin. Intermittent cultures have been positive for other flora including *Haemophilus influenzae*, *Stenotrophomonas maltophilia*, *Streptococcus pyogenes*, *Streptococcus agalactiae*, *Serratia marcescens*, *Staphylococcus pseudintermedius*, *Acinetobacter ursingii*, *Chryseobacterium indologenes*, *Pseudomonas fluorescens* group, enteric-like gram-negative bacteria, and yeast (Fig. 3). At age 13.8y, *Burkholderia cepacia* complex was detected when the patient was admitted to the emergency room for hyperglycemia. She received treatment with trimethoprim/sulfamethoxazole and levofloxacin. At age 14.1y, the patient was hospitalized with an APE and cultured *B. cepacia* complex. Meropenem and levofloxacin were administered, and the patient has remained culture-negative for *B. cepacia* complex since then.

#### Endocrine manifestations

**Fig. 4:**
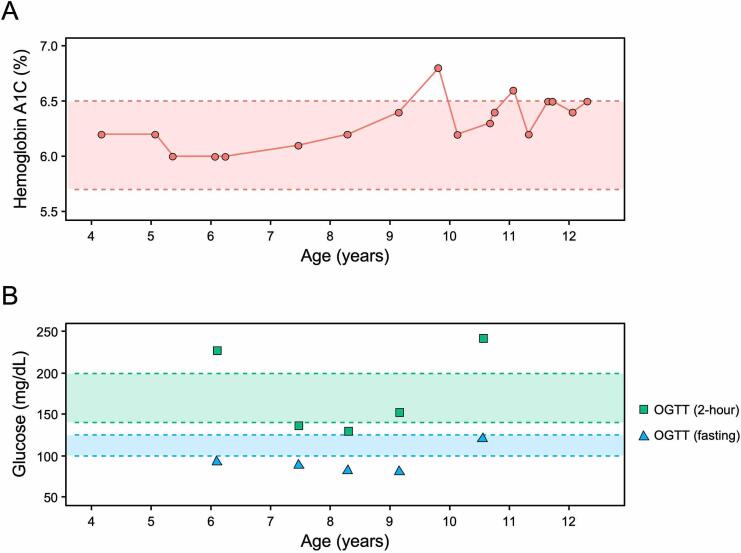
**HbA1C and OGTT results show persistent dysglycemia beginning at 4.2y.** (A) Pre-diabetic ranges are highlighted for HbA1C (5.7–6.5%; red). (B) Impaired glucose tolerance (140–200 mg/dL; green) and fasting glucose (100–126 mg/dL; blue) are indicated. From the time of CFRD diagnosis (age 6.1y) until *B. cepacia* was first detected (age 13.8y), HbA1c remained within target (<7%). For interpretation of the references to color in this figure legend, the reader is referred to the web version of this article.

Dysglycemia was first noted at age 4.2y as evidenced by an abnormal hemoglobin A1C (HbA1C) of 6.2% (Fig. 4**A**). The patient was diagnosed with CFRD at age 6.1y, with an elevated screening oral glucose tolerance test (OGTT) of 228 mg/dL (2-hour glucose) (Fig. 4**B**). At diagnosis, blood glucose levels were largely within target range (Supplementary Fig. 1), coupled with excellent weight gain and linear growth. Thus, insulin therapy was not needed. Subsequent OGTTs were normal despite pre-diabetic HbA1C levels (Fig. 4). During hospitalization for an APE at age 9.8y, hyperglycemia occurred and required temporary insulin therapy. Following hospital admission for an ensuing APE (age 10.6y), daily insulin injections were initiated. Until *B. cepacia* complex was initially cultured (age 13.8y), CFRD was well-controlled using an insulin pump with hybrid closed-loop system (average HbA1c 6.5%). Since that time, the patient’s endocrine health has steadily declined, with her most recent HbA1c measured at 13.4% (15y). Notably, many family members have type two diabetes mellitus (T2DM).

#### Gastrointestinal findings

At age 2m, the patient exhibited weight loss and chronic constipation. She was diagnosed with pancreatic insufficiency, at which point pancreatic enzyme replacement and multivitamin therapies commenced. Signs of salt wasting were not detected. During hospitalization for an APE (age 3.2y), elevated liver enzymes were noted (aspartate transaminase, 77 U/L; alanine aminotransferase, 73 U/L). However, hepatic ultrasound was normal, and liver enzymes normalized within months.

#### Other comorbidities

**Fig. 5:**
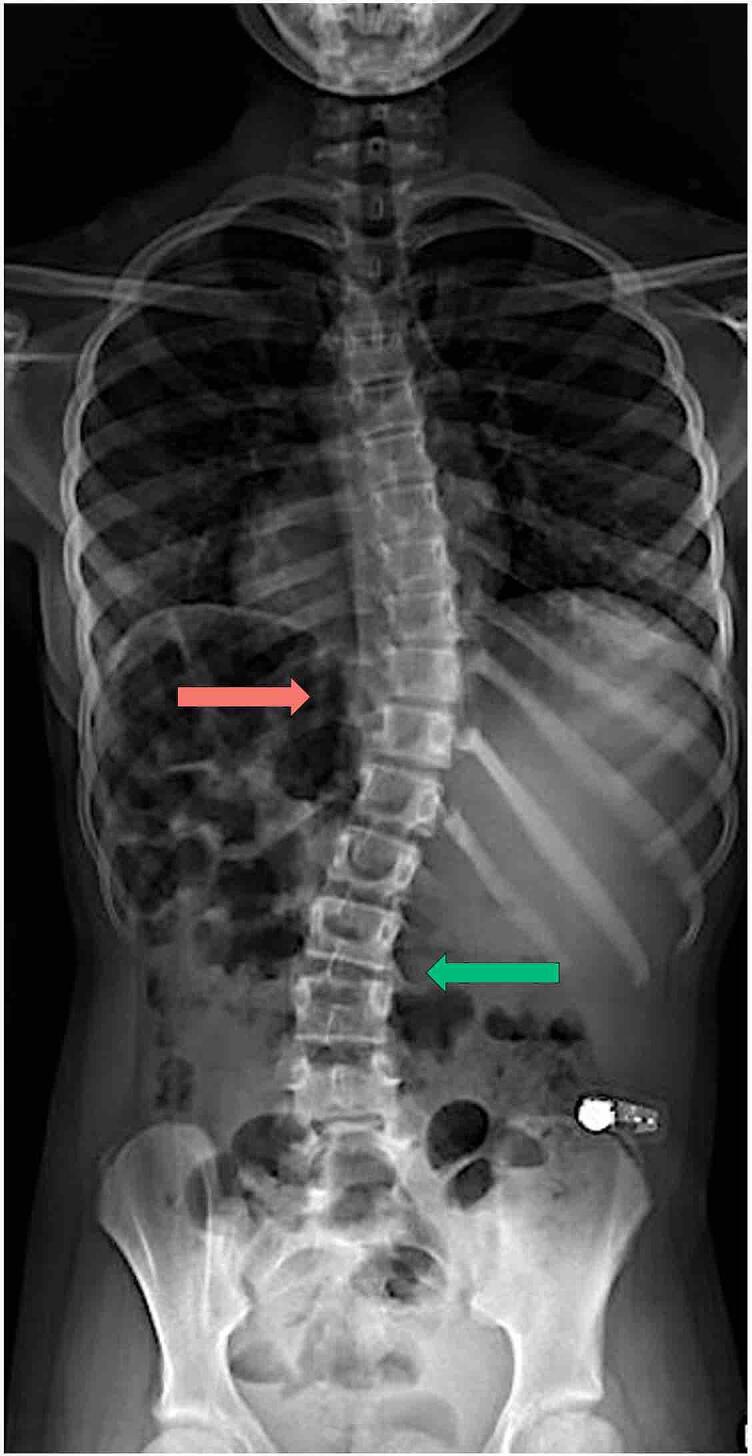
**Standing radiograph depicts mild scoliosis (age 11.9y).** Findings include dextrocurvature of the thoracic spine (19°) (red) and levocurvature of the thoracolumbar spine (21°) (green). Scoliosis was first incidentally noted from chest radiographs conducted for routine pulmonary care at age 9.2y. For interpretation of the references to color in this figure legend, the reader is referred to the web version of this article.

**Fig. 6:**
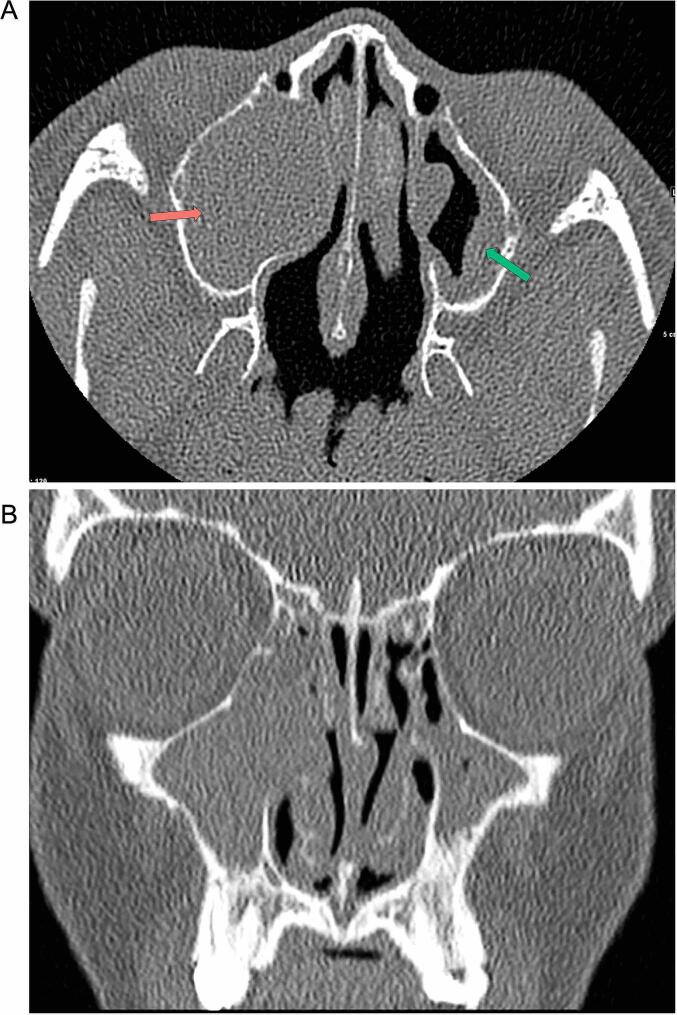
**Sinus CT at 11.3y reveals pansinusitis disease most pronounced in the maxillary sinus.** (A) Axial view is notable for mucocele causing complete opacification of the right maxillary sinus (red arrow) and mucosal thickening (green arrow). (B) Coronal view demonstrates patchy mucosal thickening throughout the ethmoid sinuses. For interpretation of the references to color in this figure legend, the reader is referred to the web version of this article.

Chest radiographs obtained for pulmonary care (age 9y) showed mild scoliosis (Fig. 5). At age 11.3y, sinus CTs revealed chronic sinusitis with nasal polyps (Fig. 6).

#### Treatments and outcomes

The patient is currently 15 years of age. Daily interventions include laxatives, intranasal steroids, sinus rinses, antihistamines, iron, pancreatic enzyme replacement, insulin, and chest physiotherapy with nebulized albuterol, 7% hypertonic saline, and dornase alfa (Table 2). Azithromycin is administered three times per week, and vitamin D3 is given once weekly. Pulmonary function decline is accelerated for an individual of the patient’s age, with FEV1 presently at 72% predicted. No digital clubbing has been observed to date. Although the patient has an automated insulin delivery device, CFRD is not well-managed (HbA1C 13.4%). It has been difficult for her to maintain therapy as prescribed due to barriers. Vitamin D deficiency persists, with 25-hydroxyvitamin D levels at 18.4 ng/mL. Dual energy x-ray absorptiometry shows normal bone mineral density (age 15y). Since age 12y, body mass index has been consistently above the 50th percentile. The patient runs track to maintain high exercise activity. She remains ineligible for CFTR modulators, since the c.2353C>T and c.1970delG variants are not on-label for these FDA-approved small molecules.

### Discussion

#### Highlighting the importance of CF endocrine, pulmonary, and genetic interactions

Incidence of CF among African Americans is ∼1:15,000 [14], [25], [26]. In Africa, lack of epidemiological data has prevented estimation of CF disease prevalence within specific countries and/or across the continent [5], [27], [28]. Sporadic reports of CF have occurred in Morocco, Algeria, Tunisia, Libya, Egypt, Sudan, Rwanda, Senegal, Cameroon, Namibia, Zimbabwe, and South Africa [27]. Although health professionals may have recognized the pathology in other countries, insufficient support for CF diagnosis, evaluation, treatment, and/or scientific publication likely contributes to under-reporting. Here, we describe a novel case of CF documented in a patient with established Gambian ancestry who encodes a new *CFTR* variant, c.1970delG. This finding is substantial, since the individual is a first-generation American citizen, whose parents emigrated from Gambia to the U.S. shortly before the patient’s birth. Therefore, it is likely that c.1970delG is indigenous to the African diaspora and represents a unique *CFTR* variant associated with the Gambian population.

The patient exhibits both common and unique features of CF disease. Atypical aspects of clinical course include appendicitis and scoliosis, which among CF populations, demonstrate frequencies of ∼1.5% [29] and 9.9–15.5% [30], respectively. Of higher concern is the patient’s unusually rapid speed of pulmonary health decline. Between the ages of 10y to 15y, her FEV1 deteriorated by 23% predicted, i.e. a rate of –3.83 percentage points per year. This change is considerably worse than a recent report showing patients 12y and older, who are served at the same CF Care Center and also not receiving modulators, exhibit a mean annualized rate of –1.92 percentage points in FEV1% predicted [31]. At age 15y, the patient’s FEV1 of 72% predicted is well below the national average of ∼99% predicted for adolescents from the same birth cohort (2008–2012) and children ages 6-18y with a similar BMI (50th percentile) [7].

Additionally, the patient’s respiratory cultures have shown comparatively rare, and significant, pathogens atypical for her age. In the 2022 U.S. CF Foundation Patient Registry, infection rates are described for CF-associated bacteria such as *P. aeruginosa* (26%), MRSA (15.6%), *S. maltophilia* (5%), and *B. cepacia* complex (1.3%) [7]. The patient in this report has cultured all of these pathogens, with *B. cepacia* complex the most surprising, as this family of bacteria is detected in less than 1% of CF adolescents aged 11-17y [7]. It has been well-established that infection with *P. aeruginosa* or *B. cepacia* complex is associated with accelerated pulmonary decline and higher mortality rate among people with CF [32], [33], [34]. Thus, these two pathogens have likely contributed to the patient’s increased frequency of APEs – i.e. six before the age of 12y. National data indicate that only ∼3% of 12-year-olds with CF have experienced two or more APEs [7].

The patient’s early-onset CFRD is also relatively unusual, as this comorbidity affects only 4.5% of the U.S. CF pediatric population [7]. Factors associated with increased risk for CFRD include female sex, pancreatic insufficiency, class I *CFTR* variants, frequent APEs, and CF liver disease [35], [36], [37]. The patient displays all of these traits with the exception of CF liver disease. Furthermore, the patient possesses a family history of T2DM. Growing evidence indicates the latter finding is a significant risk factor for CFRD, as reports have linked susceptibility genes for T2DM to CFRD [38], [39]. Prevalence of T2DM among adolescents has increased substantially in recent years, particularly within African Americans/Blacks and Hispanics/Latinos [40]. These CF populations may be subject to enhanced risk for CFRD, although robust clinical studies would be required to investigate the hypothesis.

Taken together, the patient in the current study exhibits advanced endocrine and pulmonary disease progression that is likely influenced by the afore-mentioned airway pathogens, challenges with adherence to the prescribed insulin pump-based therapy, and other factors discussed below. Numerous chronic respiratory conditions are associated with worsened lung function when diabetes is present as a comorbidity [41], [42], [43], [44]. In the context of CF, CFRD leads to increased occurrences of APEs, together with a six-fold higher risk of mortality [45], [46], [47], [48]. While a direct link between CFRD and pulmonary dysfunction has not been fully elucidated, evidence indicates that sustained elevation of blood glucose may contribute to impaired respiratory immunity [49], increased luminal bacterial growth [50], [51], and structural changes to the lungs [50], [52]. Many of these features were observed for the patient, including pulmonary bronchiectasis, frequent APEs, steadily declining FEV1, and repetitive microbial infections.

The patient’s poor clinical status is also impacted by the two severe CFTR variants encoded, c.1970delG and c.2353C>T (R785X). Both variants ultimately confer PTCs that lead to prematurely degraded CFTR mRNA and protein, resulting in no anticipated CFTR function. Although c.1970delG is a newly described variant, R785X has been established as CF-causing since 2013 [24]. R785X occurs among diverse geographic populations identified in Tunisia, Russia, China, Europe, and Ecuador [53], [54], [55], [56], [57], and is presently reported among 31 individuals in the U.S. CFTR2 database [24]. Among the U.S. patients who possess R785X and a second CF-causing variant, clinical data show the following averages: (1) 100% pancreatic insufficient; (2) sweat chloride 98 mmol/L; (3) FEV1 ∼80% predicted (age 10-20y) or ∼60% predicted (older than 20y); and (4) *P. aeruginosa* infection rate of 48%. These characteristics are nearly identical to those exhibited by the patient described herein.

Compounded with the issues noted above, the patient’s *CFTR* genotype renders her ineligible for modulators. Both c.1970delG and R785X are off-label for these clinically approved small molecules, thus omitting any potential benefits of the drugs that could be enacted upon disease progression. Patients receiving CFTR modulators have experienced improvement in clinical endpoints such as weight gain and augmented lung function, although outcomes with regard to CFRD pathogenesis have not been well-delineated [58]. Improved understanding of genetic factors and molecular mechanisms that contribute to CFRD pathogenesis, as well as impact of CFTR modulators on the pathways involved, represent important understudied areas of research.

#### Addressing racial and ethnic inequities during CF diagnosis

Despite an out-of-range NBS, the patient in this study experienced several delays in the journey to diagnosis. An initial referral for sweat chloride evaluation was provided, although this test was not performed until six weeks of age. Long-standing practice guidelines from the U.S. CF Foundation and Centers for Disease Control and Prevention recommend that sweat chloride analysis occur at 10 days of age or shortly thereafter [59], [60], [61]. At six weeks, the patient’s sweat chloride levels were well above the diagnostic threshold of 60 mmol/L, which should have resulted in a presumptive CF diagnosis but did not. The family was instead referred for a second sweat test and genetic testing, and this process encompassed approximately eight months. The extended duration was attributable to the following: (1) *CFTR* mutation analysis was not offered in Washington state at the time of the patient’s evaluation, requiring outsourcing to a laboratory on the other side of the country (Georgia); and (2) initial *CFTR* panel screening did not detect the c.1970delG and c.2353C>T variants, necessitating collection, shipping, processing, and analysis of a second blood specimen.

Notwithstanding elevated immunoreactive trypsinogen and sweat chloride concentrations, the patient did not receive a confirmed CF diagnosis until detection of two *CFTR* variants. This occurred at 10 months of age, representing a clinically significant delay [62]. The guideline for Diagnosis of CF in Screened Populations [63] states that a positive NBS and sweat chloride value of 60 mmol/L or greater confirms a CF diagnosis. Identification of *CFTR* genotype is not listed as a requirement for diagnosis but appears to have been utilized in this manner by clinicians involved in the present case. This approach is inappropriate and causes unnecessary diagnostic delays as illustrated by the patient’s experience. Prior work has established that CF diagnosis after six weeks of age significantly increases risk of irreparable damage to lung tissues [61], [64], i.e. the leading cause of mortality among CF populations. The patient’s progressive pulmonary decline likely would have been improved by standard-of-care CF therapies introduced earlier in life.

Lack of urgency was apparent during the patient’s CF diagnostic testing, which was likely impacted, at least in part, by multiple factors rooted in racial bias. Numerous reports indicate that clinical care teams incorrectly ascribe CF as a “White disease” and suggest that the diagnosis is unlikely for BIPOC individuals, even those with a positive CF NBS [65], [66], [67], [68]. This type of rhetoric is extremely harmful to patient outcomes. CF occurs in all races, ethnicities, and geographic ancestries. Regardless of demographic, aggressive “CF” management should be pursued once clinical presentation raises suspicion for the condition.

For African American/Black patients in particular, racial biases during CF NBS are influenced by a single study – cited in many CF clinical education programs – that suggests individuals with African ancestry frequently exhibit high immunoreactive trypsinogen levels without the presence of CF [69]. However, the algorithm employed in this work included second-tier testing with a repeat measurement of immunoreactive trypsinogen and genetic screening of just 32 *CFTR* variants [70]. Third-tier sweat testing was only conducted for infants who screened positive in the second-tier with consistently elevated immunoreactive trypsinogen levels (e.g. above 170 ng/mL or an ultrahigh reading in the top 0.2%) and one or two identified *CFTR* variants. The genetic panel utilized did not include numerous variants that exhibit increased prevalence among the African diaspora, such as 2307insA, A559T, D1270N, G330X, S466X, or 1812-1G>A [14]. It is therefore possible that a CF diagnosis was missed for many of the African American/Black patients without a *CFTR* variant detected during second-tier screening.

Although CF NBS has been implemented across the U.S. since 2010, states’ algorithms have differed and not always incorporated *CFTR* variant analysis. Among programs that do employ DNA testing, utilization of panels with specific *CFTR* mutations – predominantly encoded by individuals of European ancestry [22] – are known to contribute to missed and/or late CF diagnoses among BIPOC populations [10], [25], [67], [71], [72]. Recent analysis of the U.S. CF Foundation Patient Registry revealed that targeted *CFTR* screening using the ACMG 23-variant panel would not identify the full *CFTR* genotype for 56.3% of Hispanics/Latinos, 68.4% of African Americans/Blacks, 34.7% of Alaskan Natives/Indians, 74.7% of Asians, 28.6% of Hawaiian Pacific Islanders, and 49.2% of mixed races [73]. Combined with the issue that non-White individuals largely possess *CFTR* variants excluded from modulator labels [10], [14], [16], [21], [25], [74], [75], [76], [77], either due to confirmation of unresponsiveness or absence of testing, these CF communities experience distinctly elevated risk.

The ACMG has formally recognized the need for expansion of *CFTR* variant testing in NBS programs [78], which can be achieved through NGS techniques that capture mutations in agnostic fashion. Simply incorporating additional subset(s) of *CFTR* variants into panels based on predicted racial/ethnic composition among local populations would be inappropriate, as race and ethnicity are not biological variables but social constructs. Such approaches also assume accurate self-described ancestral origins provided by test subjects, which are not always known (e.g. due to genomic admixture, including consequences of historical slave trades). In addition, 2,119 *CFTR* variants have been reported [17], [23], a fraction of which have been established as disease-causing (719), varying clinical consequence (49), or unknown significance (11). Therefore, determining which *CFTR* variants are essential for inclusion presents a substantial challenge.

To reduce racial and ethnic health disparities predicated upon delays in diagnosis and treatment, we recommend that a sweat test is performed for any infant displaying high immunoreactive trypsinogen levels, even if zero *CFTR* variants are identified on a limited genetic panel. This is particularly important in the setting of individuals with non-European ancestry, which contributed to the detection of CF in the patient described in the present study. We also suggest CF NBS algorithms utilize first-tier immunoreactive trypsinogen measurement, followed by second-tier sweat chloride evaluation together with *CFTR* variant identification by NGS. *CFTR* sequencing would remove the racial/ethnic diagnostic biases intrinsic to limited *CFTR* variant panels, as these tests are comprised of mutations largely observed in people of European ancestry [14], [15], [16].

CF NBS algorithms that employ NGS have been successfully demonstrated by various programs since 2016 [79], [80], [81], [82], [83]. Blood samples undergo NGS analysis using an Illumina MiSeqDx CF Assay, for example, with accompanying software that identifies 139 CF-causing mutations. A variant-call pipeline also incorporates hundreds of additional variants classified as disease-causing [23], [24]. In cases with one CF-causing variant detected and a sweat chloride value greater than 30 mmol/L, NGS data may be re-analyzed by removing preset limitations to allow viewing of all *CFTR* variants. Thus, demonstrated feasibility of the approach supports broad utilization of NGS as a confirmatory test in CF NBS programs.

As illustrated by current findings, overcoming racial/ethnic inequities intrinsic to CF screening, diagnosis, and treatment remains a significant unmet medical need. It must be acknowledged that these processes are affected by systemic racism, which is embedded in nearly every thread of U.S. society. Meta-analysis of 293 studies conducted predominantly in the U.S. over a 30-year span (1983–2013) shows that racism is associated with poor physical and psychological health outcomes [84]. Adolescents with CF are two-to-three times more likely to develop anxiety and depression compared to the general population [85], [86], which adversely affect clinical endpoints such as quality of life, disease self-management, and survival [87], [88], [89], [90]. Mental health outcomes among youth are also negatively influenced by generational poverty, violence, and trauma, which are more prevalent among BIPOC populations due to historical and present-day structural racism [91], [92], [93], [94], [95], [96], [97], [98]. Such inequities, together with other racial/ethnic biases discussed earlier, undoubtedly shaped the patient experience described herein.

Improving equitable CF care delivery will require a sustained, multifaceted approach informed by community engaged research. Recent efforts in this arena have produced mutually reinforcing feedback from different African American/Black patient groups [68], [99], in which calls to action for CF care teams, researchers, and community organizations have been formalized. Recommended initiatives include but are not limited to the following:

(1) provide education on, and advocate for, best practices regarding unbiased CF NBS and diagnosis;
(2) make available information and resources to minoritized patients struggling to receive a CF diagnosis and/or access to care;
(3) deliver training to care teams for better understanding of racial/ethnic CF health disparities;
(4) promote culturally competent care practices that augment health equity and are personalized to the unique experiences of distinct BIPOC populations with CF;
(5) support pipelines that diversify the CF workforce, as a means to enhance the number of BIPOC physicians and other clinical staff serving minoritized patients;
(6) facilitate reoccurring community listening sessions composed of BIPOC with CF;
(7) increase BIPOC representation on committees developing CF clinical care guidelines; and
(8) feature BIPOC patients in pamphlets and other materials utilized for CF education of care providers and families.

In the future, larger-scale studies will be required to garner a comprehensive understanding of unique challenges faced by distinct groups of minoritized races and ethnicities with CF. This work will be necessary to develop interventions tailored to each population, in order to equitably improve outcomes for all people living with CF.

## Funding

This study was supported by the Cystic Fibrosis Foundation (CFF; WU20D0, WU21Q0, WU23GE0, OLIVER22A0-KB, LINNEM22QI0) and National Institutes of Health (NIH; R00HL151965, R21DK128731, UL1TR002378). CFF and NIH did not play a role in the design or conduct of the study.

## CRediT authorship contribution statement

**Malinda Wu:** Writing – review & editing, Writing – original draft, Supervision, Formal analysis, Data curation, Conceptualization, Funding acquisition. **Jacob D. Davis:** Writing – original draft, Formal analysis, Conceptualization, Data curation. **Conan Zhao:** Writing – original draft, Formal analysis, Data curation, Conceptualization. **Tanicia Daley:** Writing – review & editing, Writing – original draft, Supervision, Data curation, Funding acquisition. **Kathryn E. Oliver:** Writing – review & editing, Writing – original draft, Funding acquisition, Formal analysis, Conceptualization, Data curation, Supervision.

## Declaration of competing interest

The authors declare that they have no known competing financial interests or personal relationships that could have appeared to influence the work reported in this paper.
