# Supplementary material for: Racial inequities and rare CFTR variants: Impact on cystic fibrosis diagnosis and treatment
Source: J Clin Transl Endocrinol. 2024 Apr 20;36:100344. doi: 10.1016/j.jcte.2024.100344 (PMC11099334; doi:10.1016/j.jcte.2024.100344)
Supplement: Supplementary Data 1 [file mmc1.docx]

**
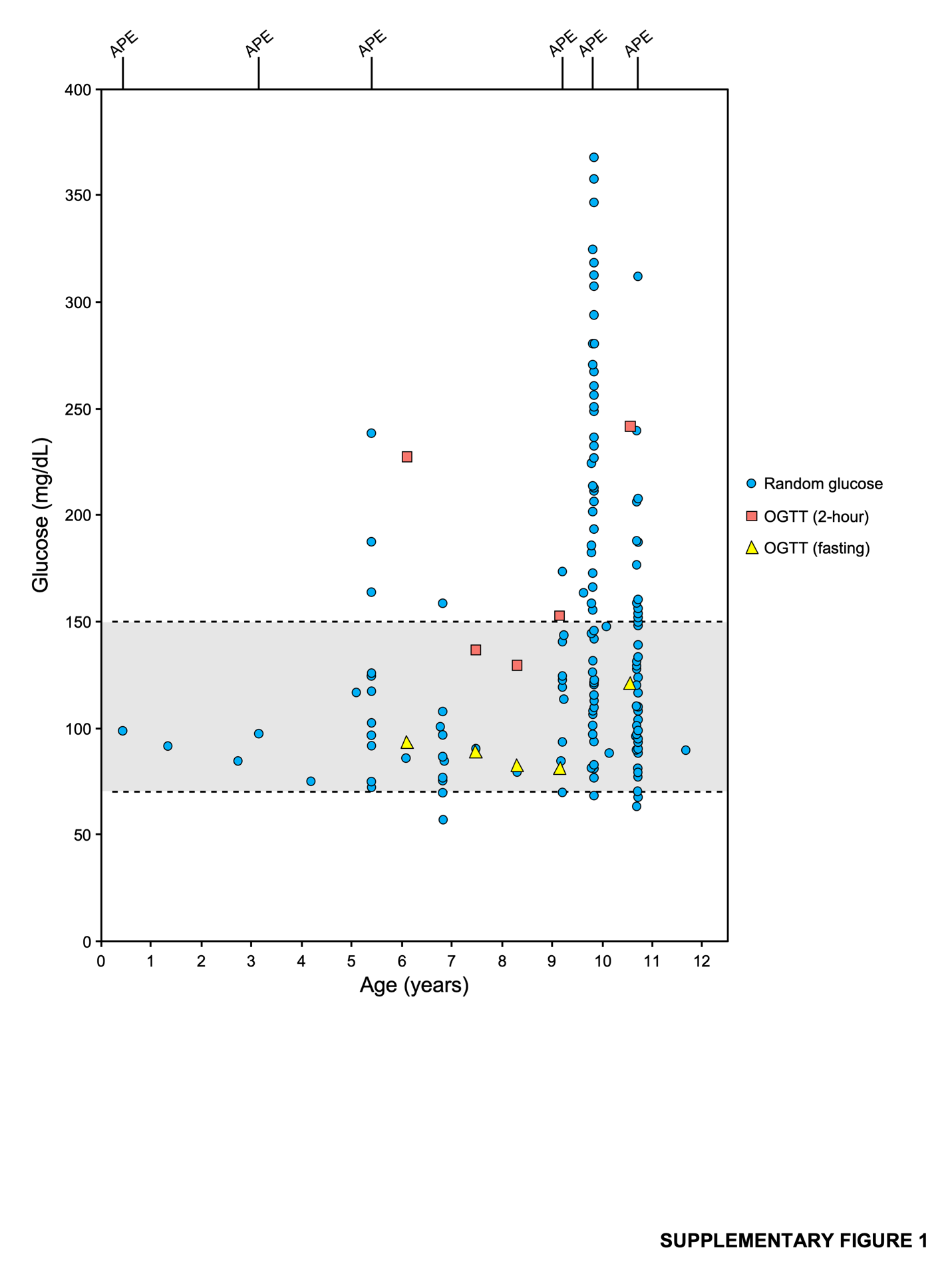
**

**Figure Legend**

**Supplementary Figure 1. Blood glucose values across lifespan.** Random glucose and OGTT results are depicted, with the CFRD goal for glycemic control (70–150 mg/dL) shaded in grey. Multiple measurements collected during hospitalizations for APEs are annotated, some of which occurred after CFRD diagnosis (age 6.1y) while on insulin therapy.
